# Metabolomics Analysis Reveals Metabolites and Metabolic Pathways Involved in the Growth and Quality of Water Dropwort [*Oenanthe javanica* (Blume) DC.] under Nutrient Solution Culture

**DOI:** 10.3390/plants12071459

**Published:** 2023-03-27

**Authors:** Kai Feng, Xia-Yue Kan, Qing Liu, Ya-Jie Yan, Nan Sun, Zhi-Yuan Yang, Shu-Ping Zhao, Peng Wu, Liang-Jun Li

**Affiliations:** 1 College of Horticulture and Landscape Architecture, Yangzhou University, Yangzhou 225009, China; fengkai@yzu.edu.cn (K.F.);; 2 Joint International Research Laboratory of Agriculture and Agri-Product Safety of Ministry of Education of China, Yangzhou University, Yangzhou 225009, China

**Keywords:** *Oenanthe javanica* (Blume) DC., gene expression, nutrient solution culture, metabolites

## Abstract

Water dropwort (*Oenanthe javanica* (Blume) DC.) is an important vegetable crop. Nutrient liquid culture has become an important cultivation method in the production of water dropwort. However, the effects of different nutrient solution cultivation methods on the growth and quality of water dropwort remains unclear. In this study, to screen the most suitable nutrient solution formula for the cultivation of water dropwort, the effects of different nutrient solution formulas (Hoagland, Cooper, Dutch greenhouse, Garden-style, Yamasaki and SCAU) on plant physiological and quality characteristics are investigated. The plant height, root length, water content (%), distribution rate of dry matter (%), chlorophyll, VC, flavonoid, total phenolic, DPPH and dietary fiber of water dropwort under different nutrient solutions were determined. According to the analytic hierarchy process (AHP) of the growth index and quality index of water dropwort under different nutrient solutions, the Yamazaki nutrient solution was considered to be the most suitable nutrient solution formula for water dropwort. To further confirm the differences of water dropwort under nutrient solution culture and soil culture, the broadly targeted metabolomics were performed. A total of 485 metabolites were detected in water dropwort under optimal nutrient solution and soil cultivation. Metabolomics analysis showed that flavonoids were the most abundant differential accumulated metabolites, and most flavonoids were up-regulated. A qRT-PCR assay indicated that the structural genes of the flavonoid biosynthesis pathway (*PAL, C4H, CHS, CHI, F3H, DFR, UFGT*) were significantly higher under the Yamasaki nutrient solution treatment. The current study provided a theoretical basis and technical guidance for the nutrient solution cultivation of water dropwort. Meanwhile, this study provides new insights into the study of flavonoids in water dropwort.

## 1. Introduction

Water dropwort [*Oenanthe javanica* (Blume) DC] is an aquatic perennial herb in the Apiaceae family, which is popular among consumers because of its high nutritional value and unique flavor [1,2]. In addition, several studies have shown that water dropwort has many medical functions, including antioxidant and antigenotoxic characteristics in human colon cancer cells [3]. Moreover, it has hepatoprotective effects [4], neuroprotective effects [5], and antiviral functions [6]. Water dropwort is always consumed as a vegetable. The edible parts of water dropwort are the fresh petioles and stems [7]. Due to its unique aroma and taste, some Southeast Asian countries use the leaves as spices for flavoring [8]. Water dropwort prefers the cold, and the suitable growth temperature is 12–24 °C [9]. Water dropwort has usually been cultivated by deep-water cultivation, shallow-water cultivation, shading cultivation and other cultivation methods [9,10]. Our previous research has found that shading cultivation was used to improve the flavor in the production of water dropwort [9]. Water dropwort has less pests and diseases, strong adaptability and is widely cultivated in the world [8,10,11,12]. The cultivation has basically realized the annual production and satisfied the annual supply of the market [13].

Soilless culture is a valid method for the horticultural production, especially in areas with severe soil degradation [14,15]. Hydroponics can be considered an engineering method for planting plants, which uses soilless growth media and optimized nutrient solutions to provide the nutrients necessary for plant growth and development [16]. Plant factories mainly use hydroponics to produce leafy vegetables through artificially formulated nutrient solutions, and the nutrients supply significantly influences plant growth in many aspects [17,18]. Compared with soil cultivation, plants cultivated in nutrient solutions have many advantages, such as a short growth cycle, fewer diseases and insect pests. The cultivation facilities can make full use of space and improve the utilization rate of land resources [19]. Nutrients are directly taken up by plants and stimulate the accumulation of different phytochemicals, such as ascorbic acid, phenolic compounds and flavonoids in plants [20,21]. Under salt stress, water dropwort has a good antioxidant defense system and shows good antioxidant capacity against salt stress [22]. Previous studies have shown that different formulations of nutrient solutions can affect the quality of hydroponics lettuce [23]. However, little information about the effect of nutrient solution formulas on water dropwort growth is available. Although there are few studies on the nutrient liquid cultivation of water dropwort, the role of water dropwort in an ecological floating bed system has been reported. The change of nutrient concentration in the process of growth and decay of water dropwort in an ecological floating bed system was studied [24].

Analytic hierarchy process (AHP) is a quantitative and qualitative analysis method [25]. It is also a reliable and adaptable multicriteria decision−making (MCDM) tool [26]. It uses a small amount of quantitative information to simplify complex problems, and applies mathematical thinking to provide simple methods to solve and analyze problems [27,28]. An analytic hierarchy process is widely used in various fields, such as urban planning, economic management, scientific research evaluation, etc. [29].

Broadly targeted metabolomics is the most commonly used metabolomics research method. Quantitative information of known and unknown metabolites in the sample to be tested can help comprehensively understand the biological phenomena [30,31,32]. Currently, metabolomics and transcriptomics were combined and analyzed in many biological studies [33,34,35]. The metabolic pathways and information involved in the growth and quality of water dropwort under nutrient solution culture was still limited. The present study investigated the effects of different nutrient solution formulas on the growth and quality of water dropwort, and screened the optimal nutrient solution formulations. Then, the metabolite differences of water dropwort under soil cultivation and nutrient solution culture with the optimal formulations were determined, using broadly targeted metabolomics. This study provides a theoretical basis and technical guidance for the high−quality and high−efficiency nutrient solution cultivation and plant industrial production of water dropwort in the future. Additionally, it provides ideas for promoting the implementation of biorefinery and circular economy strategies.

## 2. Results

### 2.1. The Effects of Nutrient Solution Culture with Different Formulations on the Growth of Water Dropwort

The growth of water dropwort under different nutrient solutions (Hoagland, Cooper, Dutch greenhouse, Garden-style, Yamasaki and SCAU) was investigated. Among them, Hoagland was used as a control group (CK). The plants of water dropwort showed distinct growth response to different nutrient solutions (Table 1). The plant height of water dropwort cultivated in Yamasaki was 31.18 cm, which was significantly higher than that of the SCAU (23.83 cm). The plant height of water dropwort cultivated in Cooper, Dutch Greenhouse and Yamasaki was slightly higher than CK. The root length of water dropwort under Cooper, Dutch greenhouse and SCAU was significantly higher than that of the other three treatments. Among them, the root length of water dropwort cultivated in Cooper was the highest, reaching 29.48 cm, 65.3% more than of CK.

The water content of water dropwort cultivated using a different nutrient solution was higher than 92%. The water content of Garden-style was the lowest. There was no significant difference among different formulas. The dry matter distribution rate of water dropwort in different treatments were higher than 72%. The highest dry matter distribution rate of water dropwort cultivated in Dutch greenhouse formula was 79.17%, which was significantly higher than that of SCAU (72.82%). There were no significant differences among CK, Cooper and Dutch greenhouse different formulas. The yield of the overground part of water dropwort was determined, and the highest yield of water dropwort cultivated in the Dutch greenhouse formula was 329.61 g, which was 8.22% higher than that of CK. The yield of water dropwort cultivated in other treatments was 1.6%, 13.0%, 1.5% and 20.5% lower than that of CK, respectively. There were significant differences among Dutch greenhouse, Garden-style and SCAU formulas.

### 2.2. The Effects of Nutrient Solution Culture with Different Formulations on the Quality Characteristics of Water Dropwort

As the petioles were the main edible parts of water dropwort, the quality characteristics of the petioles under different nutrient solutions (Hoagland, Cooper, Dutch greenhouse, Garden-style, Yamasaki and SCAU) were also determined (Table 2). The chlorophyll was higher than 44 (SPAD) in different nutrient solution formulations, and the highest value was 45.91 (SPAD) in Yamasaki. The value was slightly higher than CK in Cooper, Dutch greenhouse and Yamazaki formulations, and there was no significant difference between different formulations. The content of VC (Vitamin C) was higher than 40 μg/g, and there was no significant difference among different formulations. The highest VC content in Garden-style was 46.94 μg/g, followed by SCAU (43.43 μg/g), which was 8.3% and 0.2% higher than that of CK, respectively. 

In addition, the contents of flavonoids and total phenols in SCUA were the highest, at 1.73 mg/g FW and 11.51 mg/g FW, respectively. The flavonoid content of the Cooper formula was lower than that of CK. Moreover, other formulas were 5.8%, 9.2%, 1.1% and 13.5% higher than that of CK, respectively. The total phenol content of Cooper was lower than that of CK, while the total phenol content of other treatments was 8.3%, 6.3%, 3.8% and 29.6% higher than that of CK, respectively. There was no significant difference in DPPH free radical scavenging rate between different treatment groups, and the highest DPPH free radical scavenging rate of CK was 68.18%. While the DPPH free radical scavenging rate of other formulations was 2.2%, 14.3%, 6.9%, 9.9%, 15.7% lower than CK, respectively.

The IDF content of water dropwort cultivated in the Dutch greenhouse, Garden-style and Yamasaki formulas was slightly higher than that of CK. The highest value was 55.67% in the Dutch greenhouse formula, while the lowest value was in SCAU. The content of SDF in different formulations was significantly higher than that in CK. The SCUA was up to 8.85%, 7.7%, 26.2%, 20.0%, 15.9% and 45.1% higher than that in CK, respectively. The contents of TDF were all higher than 60%, and there was no significant difference between different formulations. The highest content of TDF was 63.37% in Dutch greenhouse formulations, and each formula was 0.7%, 3.3%, 2.3%, 2.1% and 2.8% higher than CK, respectively.

### 2.3. Comprehensive Evaluation of Growth and Quality of Spring Nutrient Solution Potted Water Dropwort Based on the Analytic Hierarchy Process (AHP) 

#### 2.3.1. Establishment of Hierarchical Structure

**Figure 1:**
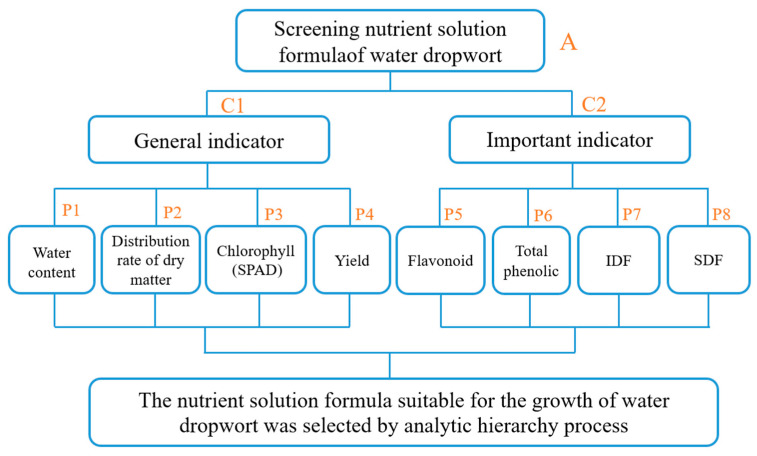
Synthesis appraisal level structural model.

According to the composition of different nutrient solution formulations, the nutrient solution formulation was selected as the target layer A, the general indicator and important indicator were used as the criterion layer C, and eight factors with great influence were selected as the index layer P according to the quality index of leafy vegetables. P1 (water content), P2 (dry matter distribution rate), P3 (chlorophyll), P4 (yield), P5 (flavonoid), P6 (total phenolic), P7 (IDF), P8 (SDF), and a comprehensive evaluation hierarchical structure model were established (Figure 1).

#### 2.3.2. Construction and Consistency Test of Judgment Matrix

Using the ratio scale quantization of 1–9, the judgment matrices of the target layer (A), the standard layer (C) and the constraint layer (P) were constructed (Table 3), which were A–C and C–P, respectively. Three judgment matrices were constructed, which were A–Ci, C1–Pi, C2–Pi, and the relative weights of each matrix were obtained. In order to ensure that the results obtained by the analytic hierarchy process are reasonable, it was necessary to test the consistency of the constructed judgment matrix. The consistency ratio CR < 0.1 of the judgment matrix could be judged as the consistency matrix. The three judgment matrices are all shown as CR < 0.1, indicating that the constructed matrix was established.

#### 2.3.3. The Final Weight Coefficient of the Comprehensive Evaluation Index

According to the index weight coefficient obtained from the judgment matrix of Table 3, the final weight coefficient of each factor of index layer P to target layer A was obtained (Table 4).

#### 2.3.4. Comprehensive Evaluation of Yield and Quality of *Oenanthe javanica* Cultivated using Different Nutrient Solution Formulas Based on Analytic Hierarchy Process

The comprehensive evaluation value was calculated by the analytic hierarchy process (AHP). The data of each index and the final weight of the index layer (Table 4) were used to calculate the comprehensive evaluation value (Table 5). Among them, the comprehensive evaluation value of water dropwort cultivated using Hoagland, Dutch greenhouse and Yamasaki were higher: 54.09, 57.68 and 54.06, respectively. Combined with the total salt of Hoagland, Dutch greenhouse and Yamasaki in Table S1, the total salt was 2160 mg/L, 1891 mg/L and 1382 mg/L, respectively. The Yamasaki formula was more suitable for the cultivation of water dropwort ‘Fu qin No. 1‘.

### 2.4. The Profiling of Metabolites 

According to the analysis of the growth and quality indexes of water dropwort treated with different formulations, Yamasaki was the most suitable formula for nutrient solution cultivation of water dropwort. In order to further reveal the effects of nutrient solution culture on the accumulation of metabolites, a broadly targeted metabolomics tests were carried out on water dropwort with soil culture (CK) and Yamasaki nutrient solution (NK). A total of 485 metabolites were identified from water dropwort cultivated under CK and NK, including 104 flavonoids, 76 phenolic acids, 61 amino acids and derivatives, 54 lipids, 40 organic acids, 40 nucleotides and derivatives, 30 lignans and coumarins, 24 alkaloids, two terpenoids, one tannin and 53 others (Table 6). 

### 2.5. Validation of the OPLS-DA Model 

PCA results show the trend of metabolome separation between groups, indicating whether there is a difference in metabolomes between sample groups. In the PCA score diagram, the two groups of samples were clearly separated without crossing areas, and the first two principal components, PC1 and PC2, accounted for 40.03% and 25.04%, respectively (Figure S1a). After adding quality control samples, PC1 accounted for 36.96% and PC2 accounted for 22.56% (Figure S1b). According to the OPLS−DA model, 485 metabolomic data were analyzed. The left side of the confidence interval was the water dropwort sample cultivated in soil, and the right side of the confidence interval was the water dropwort sample cultivated in a nutrient solution. The two samples had a good discrimination effect (Figure S2a). OPLS−DA analysis obtained two principal components, whereby the PC1 contribution rate accounted for 40.8%, PC2 contribution rate accounted for 17.8%, R2X = 0.567, R2Y = 0.999, Q2 = 0.866, among which Q2 > 0.5 is the effective model, Q2 > 0.9 is an excellent model, and the model built is relatively good. The 200 permutation experiments were performed to verify the OPLS−DA model (Figure S2b). Q2 and R2Y of the original model are red and blue horizontal lines, respectively. Q2‘and R2Y’ are models after Y replacement, represented by red and blue dots. The red and blue dots are not higher than the corresponding red and blue lines, and the model after Y replacement is smaller than the original model, indicating that the model is meaningful. VIP value analysis can be used to screen the differential metabolites of samples.

### 2.6. Differential Metabolite Screen

**Figure 2:**
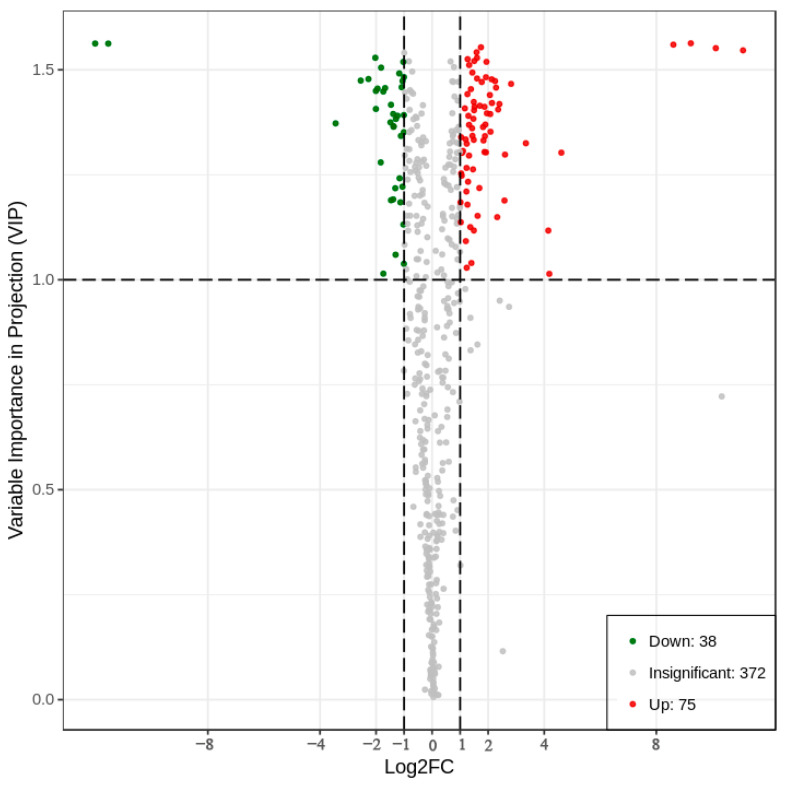
Volcanic map of differential metabolites of water dropwort under soil culture (CK) and Yamasaki nutrient solution (NK). Note: each point on the volcanic map represents a metabolite, and the abscissa represents the logarithm of the quantitative difference multiple of a metabolite in the two samples. The y−coordinate represents the VIP value. Red represents the metabolites with upregulated differential expression, green represents the metabolites with down−regulated differential expression and gray represents the detected metabolites with no significant differences.

Fold change and variable importance in project (VIP) of the OPLS−DA model were combined to screen differential metabolites. The criteria for screening included a fold change value of ≥ 2 or ≤ 0.5, and a VIP value of ≥ 1. A total of 113 differential metabolites were screened from the water dropwort cultivated with CK and NK, accounting for 23.3% of the total metabolites, including 75 up−regulated metabolites and 38 down−regulated metabolites (Figure 2). The 113 differential metabolites were divided into 10 categories, including flavonoid amino acids and their derivatives, other organic acids, phenolic acid lipids lignans, and coumarin amino acids and their derivatives, alkaloid terpenes. The results indicated that flavonoids accounted for 20% of all differential metabolites, which were the most abundant differential metabolites (Figure S3). 

### 2.7. Analysis of Differential Metabolites 

**Figure 3:**
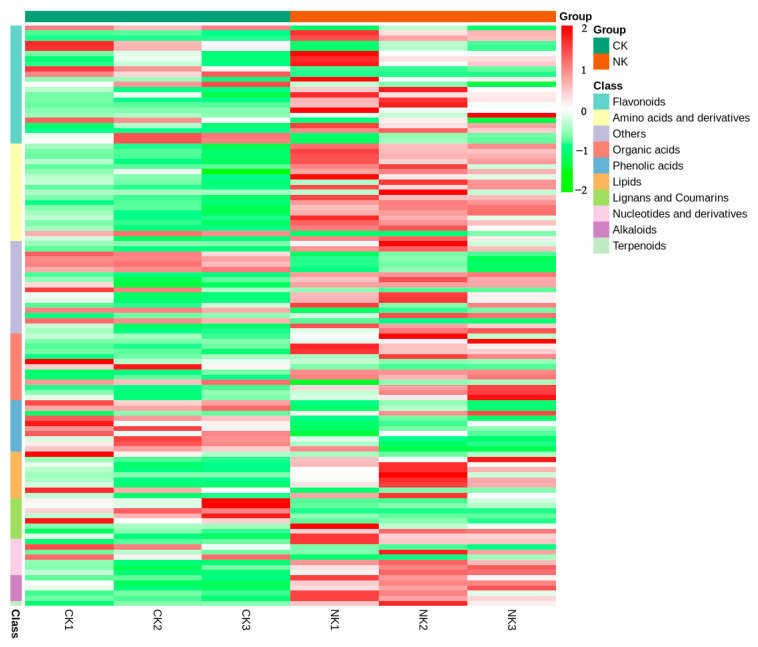
Differential metabolite clustering heat map of water dropwort under soil culture (CK) and Yamasaki nutrient solution (NK).

**Figure 4:**
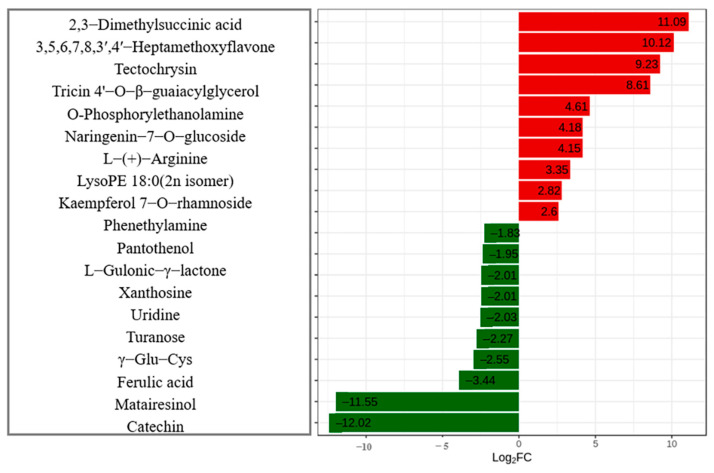
Differential metabolites bar chart of water dropwort under soil culture (CK) and Yamasaki nutrient solution (NK). Red represents up−regulated differentially expressed metabolites and green represents down−regulated differentially expressed metabolites.

In this study, unit variance scaling (UV) was used to analyze the differential metabolites, and the heat map was drawn (Figure 3). The heat map simply and intuitively showed that there were 38 down−regulated differential metabolites with reduced relative content in the NK compared with the CK, accounting for 33.6% of the differential metabolites, of which 75 were up−regulated, accounting for 66.4% of the differential metabolites. The metabolites were analyzed qualitatively and the differential multiple changes of the quantitative information of metabolites were compared with the grouping of samples. After log−2 treatment, the top 20 differential metabolites were selected (10 up−regulated and 10 down−regulated), the largest up−regulated metabolite was 2, 3−dimethyl−butylacid (11.09), and the smallest down−regulated metabolite was O−feruloyl 4−hydroxylcoumarin (−12.02) (Figure 4). Among the flavonoids with the largest number, 3,5,6,7,8,3, 4−heptamethoxyflavone was the most up−regulated metabolite with the largest difference multiple (10.12), and catechin was the least down−regulated metabolite (−11.55). Among the top 20 differentially expressed metabolites, five flavonoids were up−regulated and only one was down−regulated.

### 2.8. KEGG Annotation and Enrichment Analysis of Differential Metabolites 

**Figure 5:**
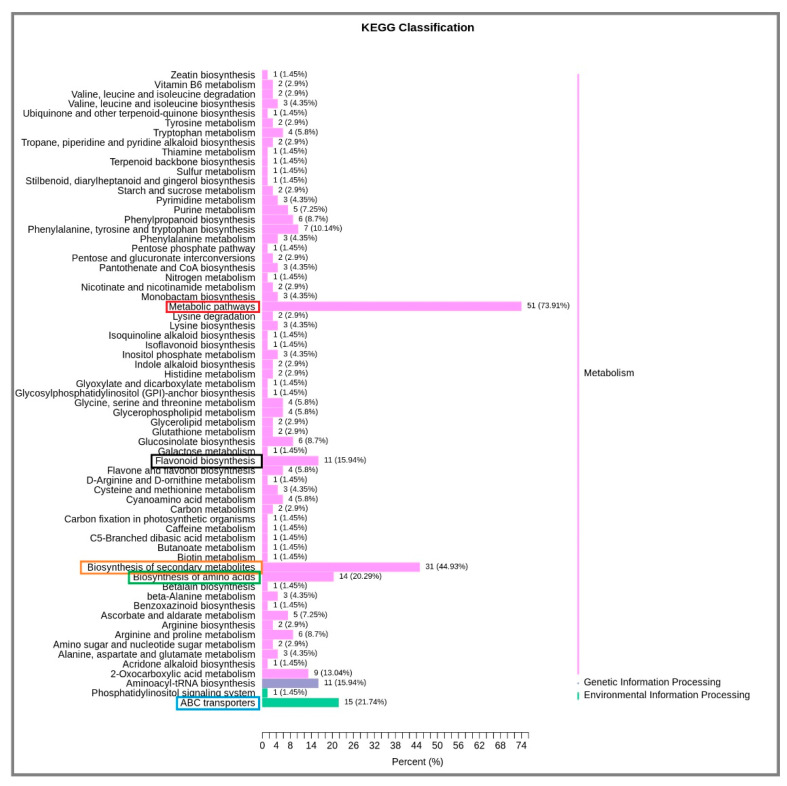
KEGG classification of differential metabolites of water dropwort under soil culture (CK) and Yamasaki nutrient solution (NK). Pink represents pathways related to metabolism, purple represents pathways related to genetic information processing, and green represents pathways related to environmental information processing.

**Figure 6:**
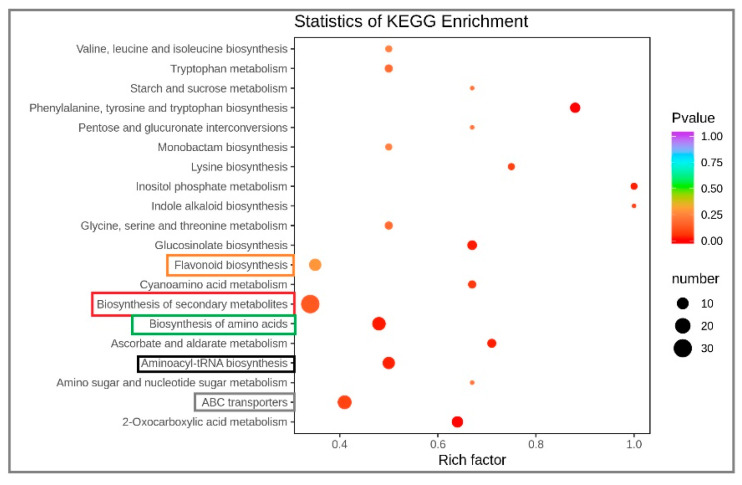
KEGG pathway enrichment of differential metabolites of water dropwort under soil culture (CK) and Yamasaki nutrient solution (NK). The abscissa indicates the rich factor corresponding to each path, the ordinate is the path name, the color of the point is the p−value, and the redness level indicates that the enrichment is more significant. The size of the dots represents the number of differential metabolites enriched.

The different metabolites interact in an organism to form different pathways. The Kyoto Encyclopedia of Genes and Genomes (KEGG) database was used to annotate and further analyze the annotation results for classification and enrichment. Fifty−one metabolic pathways were involved, accounting for 73.91% of the total number. Biosynthesis of 31 secondary metabolites accounted for 44.93% of the total. The biosynthesis of ABC transport amino acids, flavonoid biosynthesis and aminoyl−TRNA biosynthesis were of 15, 14, 11 and 11 metabolites, respectively, accounting for 21.74%, 20.29%, 15.94% and 15.94% of the total number (Figure 5). There were 20 main metabolic pathways for the distribution of differential metabolites. The results demonstrated that 20 metabolic pathways of differential metabolites were mainly involved in secondary metabolite biosynthesis, amino acids biosynthesis, flavonoid biosynthesis metabolic pathway, ABC transport, amino acid biosynthesis and aminoacyl−trNA biosynthesis (Figure 6). 

### 2.9. The Expression Levels of the Gene Involved in the Flavonoid Metabolism Pathway

**Figure 7:**
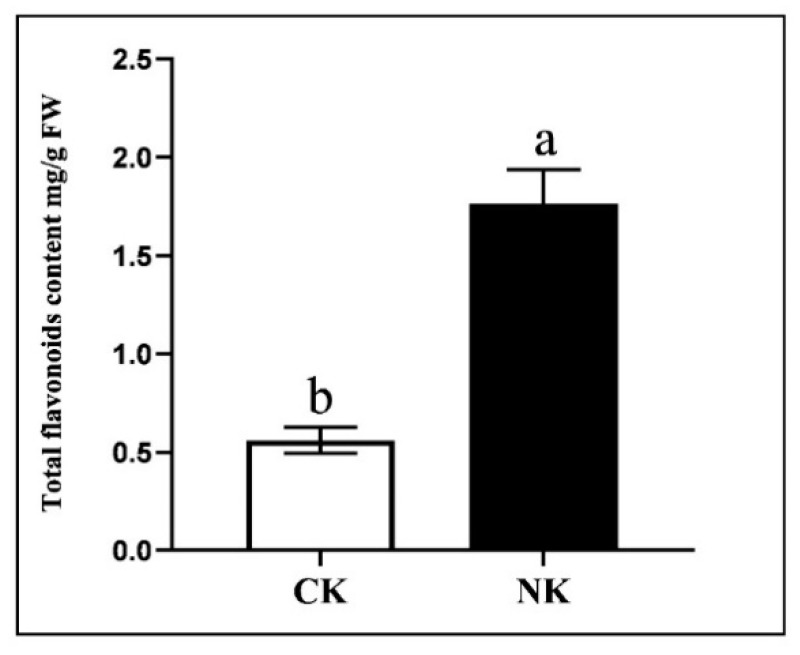
The total flavonoid content of water dropwort under soil culture (CK) and Yamasaki nutrient solution (NK). Different letters indicate a significant difference in the total flavonoid content of water dropwort between different treatments, at the 5% level. Error bars show the standard deviation (SD) of three biological replicates.

**Figure 8:**
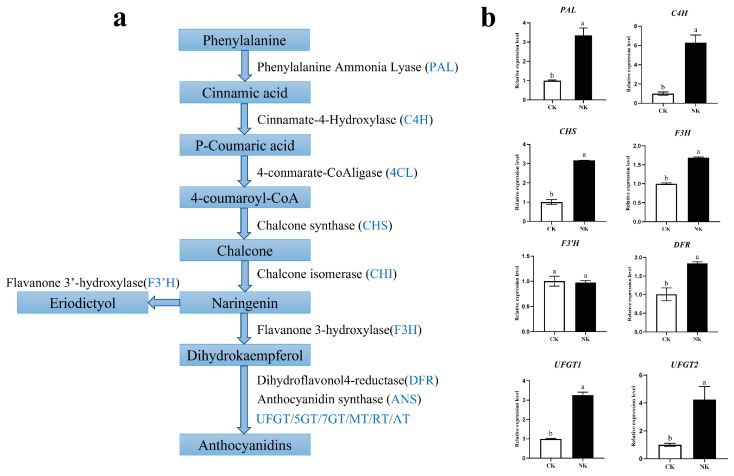
The expression levels of the gene involved in the flavonoid metabolism pathway. (**a**) Schematic diagram of the flavonoid synthesis pathway. (**b**) qRT-PCR analyzed the relative expression of flavonoid pathway structural genes. Different letters indicate a significant difference in the relative expression of the flavonoid pathway structural genes of water dropwort between different treatments, at the 5% level. Error bars show the standard deviation (SD) of three biological replicates.

In the study, 113 differential metabolites were determined, and flavonoids were the main differential metabolites. As shown in Figure 7, the total content of flavonoids under the Yamasaki nutrient solution (NK) was significantly higher than that in soil culture (CK). The biosynthesis of flavonoids is a complex process that is gradually formed under the catalysis of a series of enzymes, including early biosynthetic genes (*PAL, C4H, 4CL, CHS, CHI*) and late biosynthetic genes (*F3H, F3′H, DFR, ANS, UFGT, ANR*) [36,37] (Figure 8a). In order to further investigate the expressions of key genes involved in the flavonoid biosynthesis pathway, the qRT-PCR assay of water dropwort under NK with CK was performed. The expressions of the most structural genes (*PAL, C4H, CHS, CHI, F3H, DFR, UFGT*) in the flavonoid pathway were significantly up−regulated under NK (Figure 8b). These results suggest that the expressions of these key genes were correlated with the flavonoid contents in water dropwort under different cultivation. 

## 3. Discussion

It is well known that water dropwort is a popular vegetable, rich in vitamins, proteins, dietary fibers and other nutrients [38]. Nutrient liquid cultivation plays a very important role in improving vegetable planting efficiency in limited soil agricultural systems [39]. Different nutrient solution formulations have different effects in the growth and quality of water dropwort, probably due to the uptake and preference of mineral element absorption [20]. According to the analytic hierarchy process (AHP), comprehensively considering the effects of different nutrient solution cultivation on the growth and quality of water dropwort, the Yamazaki nutrient solution was considered to be the most suitable classic nutrient solution formula. The chlorophyll synthesis is associated with different N sources, which are ultimately correlated with the photosynthesis rate and determine the final crop yield [40]. In this study, the leaf chlorophyll value (SPAD) under the Yamazaki nutrient solution culture was higher than that under other nutrient solution treatments. In the results of this study, the yield and quality of water dropwort cultured with the Yamazaki nutrient solution were better. This might be related to the ratio of NH_4_^+^/ NO_3_^—^ in the Yamazaki formula. The application of 25% NH_4_^+^/75% NO_3_^—^ ratio increased the shoot fresh biomass of lettuce in hydroponic cultivation systems [41]. In terms of the yield of water dropwort, Hoagland, Cooper, Dutch greenhouse and Yamasaki four treatments were significantly higher than those of Garden-style and SCAU treatments. A high K^+^/Na^+^ ratio also plays a vital role in maintaining membrane potential, as well as osmotic and turgor pressures [42,43]. It also helps in enzyme activation and tropisms. Soil salinity enhances the Na^+^ and Cl^—^ contents in plants, which then increases the Na^+^/K^+^ ratio, which ultimately affects regular ionic activities in plants [44]. The difference of ions in nutrient solution affects the nutrients absorbed by the root of water dropwort, which may be the main reason for the difference in its yield [22]. Using six kinds of culture nutrient solutions, the growth and quality indexes of water dropwort were determined. The Yamasaki culture solution was the best hydroponics nutrient solution for water dropwort. The current study aims to determine the effects of different nutrient solution cultivation on the growth and development of water dropwort. The growth curve of water dropwort and changes of nutrient solution during various growth stages will be our research focus in the future. To further explore the effect of different cultivation methods on the accumulation of metabolites, metabolomics analysis was conducted on the water dropwort cultivated with soil culture and the optimal nutrient solution formula.

Based on the broadly targeted metabolomics method, the metabolic changes of water dropwort cultivated in soil and nutrient solution were studied. A total of 485 metabolites were detected and 113 different metabolites were screened. The overall performance was up−regulated. From the level of metabolomics further validated, the quality of water dropwort cultivated in nutrient solution was better than that of soil cultivation. Metabolomics analysis showed that the flavonoids were the most abundant differential metabolites among water dropwort under nutrient solution cultivation and soil cultivation. The relative content of flavonoid metabolites accumulated continuously during the development of *E. ferox* seeds, and reached the highest at T30 [34]. In transcriptome, the expression of key genes in the flavonoid pathway, such as *PAL, CHS, F3H, FLS* and *ANS,* was the highest in T30, which was consistent with the trend of metabolites [34]. Flavonoids are important secondary metabolites and are widely studied in plants [45], which were divided into flavonols, flavones, dihydroflavonoids, flavonosides, dihydroflavonols and so on [46,47]. They play an important role in plant growth and development and resistance to stress, such as pigmentation, seed coat development, plant pollination, and resistance to UV radiation and pathogen infection [48,49,50]. Alongside that, flavonoids are beneficial to human health due to their antioxidant, anticancer and antimicrobial biological activities [51,52,53]. 

Flavonoids originate from the phenylpropane pathway, and the differential expressions of structural genes caused the changes of flavonoid accumulation in plants [54]. *PAL* is the first rate−limiting enzyme in the phenylpropane metabolic pathway which controls the transformation of primary metabolites to secondary metabolites [55]. *CHS* is the first rate−limiting enzyme in the flavonoid pathway that catalyzes the formation of chalcone from a molecule of coumaroyl−COA and a molecule of 3 malonyl−COA [56,57,58]. *FLS* is a key enzyme in the biosynthesis of flavonols, while *DFR* and *ANS* are key enzymes in the biosynthesis of anthocyanins [59]. In the study, the expression of the key genes (*PAL, C4H, CHS, CHI*, *F3H, DFR, UFGT*) was significantly higher under the Yamasaki nutrient solution treatment. The different fold values of flavonoids in nutrient solution cultivation of water dropwort were large, and the specific pharmacological effects can be further studied based on metabolomics. 

Previous studies have found that the unique aroma of water dropwort is related to the accumulation of secondary metabolites, including terpenoids, alcohols, etc. [7,9,60]. Our previous research has found that shading cultivation was used to improve the flavor in the production of water dropwort. The terpenoid biosynthesis genes were down−regulated under the shading treatment [9]. In addition, GC−MS technology was used to determine and analyze the volatile substances related to the flavor of water dropwort [7]. The β−caryophyllene synthetase gene *OjTPS1*, a characteristic flavor substance of water dropwort, was screened. We have completed the preliminary functional analysis of the *OjTPS1* gene on the regulation of β−caryophyllene synthesis [7]. In this study, terpenoids were also included in the differential metabolite classification of water dropwort cultivated in nutrient solution and soil. Moreover, KEGG enrichment analysis of differential metabolites showed that many metabolites were distributed within the terpenoid anabolic pathways. These results may provide new ideas for further research on the flavor of water dropwort. 

## 4. Materials and Methods

### 4.1. Plant Material 

The plant material used in this study was *O. javanica* (Blume) DC. cultivar ‘Fu qin No. 1′. The seedlings of water dropwort, with a plant height of about 5 cm, were selected for experiment.

### 4.2. Hydroponic System

The experiment was carried out in the aquatic vegetable experimental field base of Yangzhou University in spring, and the temperature and light amount equality were under natural conditions. Hydroponics was carried out in the growth tray (380 mm × 275 mm × 140 mm). Nutrient solution (12 L) was added to the growth tray. Each frame contained 15 plants of water dropwort and the plants were fixed with polystyrene foam board. The roots were penetrated into the nutrient solution and the height of the nutrient solution was 10 cm. The cultivation with different nutrient solution formula was conducted with three biological replicates.

### 4.3. The Nutrient Solution Culture of Water Dropwort

The nutrient solution with six different compositions (Hoagland, Cooper, Dutch greenhouse, Garden-style, Yamasaki and SCAU) of macronutrients was applied on water dropwort growth in the experiment (Table S1) [61]. The micronutrients of different nutrient solutions were set based on the general formula (Na_2_Fe·EDTA 20 mg/L, H_3_BO_3_ 2.86 mg/L, MnSO_4_·4H_2_O 2.13 mg/L, ZnSO_4_·7H_2_O 0.22 mg/L, CuSO_4_·7H_2_O 0.08 mg/L, (NH_4_)_6_Mo_7_O_24_ 0.02 mg/L). 

### 4.4. Determination of Biomass Indexes 

Samples were collected 35 days after planting. Three strains were selected randomly for index determination. Freshly retrieved samples were first cleaned with steaming water, and then dried with absorbent paper. Plant height and root length were measured, weighed, and the fresh weight of aboveground and underground parts was recorded. Measurement of water content and distribution rate of dry matter of the samples were carried out, after drying to a constant weight using an electro−thermostatic blast oven.

### 4.5. Determination of Physiological Indexes 

#### 4.5.1. Measurement of Chlorophyll

Chlorophyll was determined by SPAD−502 Plus chlorophyll analyzer (Konica Minolta, Tokyo, Japan). SPAD−502 Plus rapid chlorophyll analyzer was used to measure the chlorophyll content of petiole tip leaflets, and 15 values were measured each time and the average value was taken.

#### 4.5.2. Measurement of Vitamin C (VC)

The VC content was determined according to the instructions of the assay kit. The vitamin C (VC) content test kit (colorimetric method) was purchased from Nanjing Jiancheng Bioengineering Institute (Nanjing, China). VC was determined by colorimetry. Briefly, 0.15 mL of the sample was dissolved in 0.45 mL of reagent 1 application solution, mixed well and allowed to sit for 15 min. The sample was centrifuged for 10 min and the upper clear liquid was the supernatant. Then, reagent 1 application solution 0.4 mL, reagent 2 application solution 0.5 mL, reagent 3 application solution 1 mL and reagent 4 application solution 0.25 mL were added to the blank tube. The 6 µg/mL VC standard application solution 0.4 mL, reagent 2 application solution 0.5 mL, reagent 3 application solution 1 mL and reagent 4 application solution 0.25 mL were added to the standard tube. The supernatant 0.4 mL, reagent 2 application solution 0.5 mL, reagent 3 application solution 1 mL and reagent 4 application solution 0.25 mL were added to the determination tube. And were fully mixed on a 37 °C water bath for 30 min. The reagent 5 application solution 0.1 mL was added to the tube, fully mixed and allowed to stand for 10 min. The absorbance of each tube was measured with a spectrophotometer, with a wavelength of 536 nm and an optical diameter of 1 cm (or 0.5 cm). 

#### 4.5.3. Measurement of Flavonoids, Total Phenolic, DPPH

The samples of water dropwort were placed in a constant-temperature drying oven, dried at 105 °C for 30 min, and stored at 60 °C. After drying, the dry weight was weighed and ground into a powder, which was used for the determination of flavonoids, total phenolic and DPPH free radical scavenging rate. The flavonoid, total phenolic and DPPH content of water dropwort leaves ware determined in accordance with the protocol of Plant Flavonoids Test kit (Suzhou Comin Biotechnology Co., Ltd., Suzhou, China) [62].

Flavonoids were determined by spectrophotometry. The sample was dried to a constant weight, crushed and passed through a 40-mesh sieve. About 0.02 g of sample was dissolved into 2 mL of 60% ethanol extract, and shaken at 60 °C. Then, 540 µL of distilled water and reagent 1 application solution 30 µL were added to the blank tube. Regarding the sample solution to be tested, 540 µL and reagent 1 application solution 30 µL were added to the determination tube, fully mixed and allowed to stand at 25 °C for 6 min. Reagent 2 application solution 30 µL was added to each test tube, fully mixed and allowed to stand at 25 °C for 5 min. Finally, reagent 3 application liquid 400 µL was added to each tube, fully mixed and allowed to stand at 25 °C for 15 min. The absorbance was determined at 510 nm. Δ A = Δ A determination−A blank. Total phenolic and DPPH content were also determined by spectrophotometry. The specific method is similar to the above flavonoid determination method.

#### 4.5.4. Measurement of Dietary Fiber 

The solvent was 0.06 mol/L hydrochloric acid, the ratio of solid to liquid was 1:25 (g/mL), the extraction time was 90 min and the extraction temperature was 90 °C. At room temperature, it was centrifuged at 3000 rmp for 20 min and the supernatant reserved. The residue was washed with distilled water, centrifuged at room temperature at 3000 rmp for 20 min, and the washing solution was kept. The residue was insoluble dietary fiber (IDF) after drying. The extraction solution was the combined supernatant and washing solution. A 95% ethanol solution of 4 times the volume of the extraction solution was added and mixed, and left for 1 h at room temperature. At room temperature, this was centrifuged at 5000 rmp for 20 min, the filtrate was discarded and the precipitate dried to become soluble dietary fiber (SDF). Total dietary fiber (TDF) is the sum of soluble and insoluble dietary fiber.

### 4.6. Analysis Methods and Steps of the Analytic Hierarchy Process (AHP) 

Eight characteristics (water content, dry matter distribution rate, chlorophyll, yield, flavonoid, total phenolic, IDF, SDF,) were selected to form a comprehensive evaluation index set. The hierarchical structure model was established. The judgment matrix in each level was constructed and consistency test was carried out to calculate the weight of the selected index. The original data were transformed to calculate the comprehensive evaluation set. 

The analytic hierarchy process was used to determine the weight of each index. All the judgment matrices in each level were constructed, and the maximum eigenvalue λ_max_ and the corresponding eigenvector W = (W_1_, …, W_8_), were calculated by using the Matlab software [25]. The consistency ratio CR was calculated according to the formulas CI = (λ_max_ − n)/(n − 1) and CR = CI/RI (where the average random consistency index RI referred to the value given by Saaty [25]). When CR < 0.1, it was considered that the consistency of the judgment matrix is acceptable. The calculation method of the final weight coefficient of the comprehensive evaluation index is to multiply the weight coefficient of each evaluation index (P) relative to the standard layer (C), by the weight coefficient of the standard layer (C) relative to the target layer (A). Finally, the weight value of each evaluation index (P) to the target layer (A) is calculated.

### 4.7. Metabolomics Analysis

#### 4.7.1. Sample Extraction

To further explore the effect of different cultivation methods on the accumulation of metabolites, the metabolomics analysis was conducted on the water dropwort cultivated in the optimal nutrient solution formula and soil culture. After harvesting, water dropwort under soil culture (CK) and nutrient solution culture (NK) were selected for broadly targeted metabolomics detection. The freeze-dried water dropwort leaf was crushed using a mixer mill (MM 400, Retsch, Germany) with a zirconia bead, for 1.5 min at 30 Hz. Next, 100 mg of powder was weighed and extracted overnight at 4 °C with 0.6 mL of 70% methanol aqueous solution. After centrifugation at 10,000× *g* for 10 min, the extracts were absorbed (CNWBOND Carbon−GCB SPE Cartridge, 250 mg, 3 mL; ANPEL, Shanghai, China) and filtrated (SCAA−104, 0.22 μm pore size; ANPEL, Shanghai, China), before UPLC−MS/MS analysis [63].

#### 4.7.2. UPLC Conditions

The sample extracts were analyzed using an UPLC−ESI−MS/MS system (UPLC, Shim−pack UFLC SHIMADZU CBM30A system; MS, Applied Biosystems 4500 Q TRAP) [64]. The analytical conditions were as follows: UPLC column, Waters ACQUITY UPLC HSS T3 C18 (1.8 µm, 2.1 mm × 100 mm); the mobile phase consisted of 0.04% acetic acid aqueous solution solvent A and 0.04% acetic acid acetonitrile solvent B; the gradient procedure was used for sample measurement, and the initial conditions were 95% A and 5% B. Within 10 min, the combination of linear gradient to 5% A, 95% B, 5% A and 95% B were maintained for 1 min. 

#### 4.7.3. ESI−Q TRAP-MS/MS

LIT and triple quadrupole (QQQ) scanning were carried out on three four-pole linear ion trap mass spectrometer (Q TRAP), API 4500 Q TRAP UPLC/MS/MS System. The system was equipped with an ESI Turbo ion spray interface, which operates in positive and negative ion modes and is controlled by the Analyst 1.6.3 software (AB Science). Instrument tuning and mass calibration were performed with 10 and 100 μmol/L polypropylene glycol solutions in QQQ and LIT modes, respectively [65]. When the collision gas (nitrogen) was set to 5 psi, the QQQ scan was obtained through an MRM experiment. On the basis of further optimizing DP and CE, DP and CE of single MRM transition were completed. A specific set of MRM transitions were monitored for each period, according to the metabolites eluted within this period [64].

#### 4.7.4. Qualitative and Quantitative Metabolites

High-resolution mass spectrometry AB SCIEX TripleTOF 6600 and AB SCIEX QTRAP 4500 were used for qualitative detection and quantification of mixed samples in widely target metabolomics analysis, respectively [66]. Then, the identification was conducted by match of mass spectrum to reference library MetWare database (MWDB). MWDB was constructed based on the standard compounds or public database, such as METLIN [65,67]. Qualitative parameters contained an accurate mass of metabolites, MS2 fragments, MS2 fragments isotope distribution and retention time (RT). The secondary spectrum and RT of the metabolites in the project samples were compared with MWDB. The MS tolerance and MS2 tolerance were set to 20 ppm and the RT offset did not exceed 0.2 min [64]. The quantitative analysis of metabolites was completed by triple quadrupole mass spectrometry multiple-reaction monitoring (MRM) analysis. After obtaining the metabolite spectrum analysis data of different samples, the mass spectrum peaks of all substances were integrated, and the mass spectrum peaks of the same metabolite in different samples were integrated and corrected [68]. Mass spectrometry data were processed using the software Analyst 1.6.3.

#### 4.7.5. Sample Quality Control Analysis

Repeatability of metabolite extraction and detection can be determined by overlapping display and analysis of the total ion current diagram (TIC diagram) of precise spectrum detection and analysis of different QC samples. Quality control samples (QC) were prepared by mixing sample extracts. The retention time and peak intensity of metabolite detection were consistent, indicating that the signal stability of mass spectrometry was good when detecting the same sample at different times (Figure S4). Based on the local metabolic database, the metabolites of the samples were analyzed qualitatively and quantitatively by mass spectrometry. The MRM metabolite detection multipeak diagram shows the substances that could be detected in the sample. Each mass spectral peak with different colors represents a metabolite detected. The characteristic ions of each substance were screened by triple four-stage bars, and the signal intensity (CPS) of the characteristic ions was obtained in the detector. MultiaQuant software (3.0) was used to open the mass spectrometry file of samples, and the integration and correction of chromatographic peaks were carried out. The peak area of each chromatographic peak represented the relative content of the corresponding substance. Finally, all chromatographic peak area integral data were derived and saved. 

#### 4.7.6. Differential Metabolite Selected

Principal component analysis (PCA) is a multidimensional statistical analysis method for unsupervised pattern recognition. The transformed variables are called principal components [69]. OPLS−DA is a multivariate statistical analysis method with supervised pattern recognition, that can maximize the distinction between groups and is helpful for finding different metabolites [70]. Based on the results of OPLS−DA, the metabolites of different varieties or tissues can be preliminarily screened from the variable importance in project (VIP) of multivariate analysis OPLS−DA model [71]. Meanwhile, the folding VIP value and OPLS−DA model were used to screen the differential metabolites. Screening criteria: discount ≥ 2, discount ≤ 0.5, VIP ≥ 1.

#### 4.7.7. KEGG Functional Annotation and Enrichment Analysis of Differential Metabolites

The KEGG (Kyoto Encyclopedia of Genes and Genomes) database is helpful for researchers to study genes, expression information and metabolite content as a whole network. Differential metabolisms interact with each other in organisms to form different pathways. The differential metabolites were annotated and presented using the KEGG database [72]. KEGG pathway enrichment was carried out according to the results of differential metabolites, where the rich factor was the ratio between the number of differentially expressed metabolites in the corresponding pathway and the total number of metabolites detected and annotated by this pathway. The larger the value, the greater the enrichment degree. There were many types of metabolites, and the substances gradually increased. However, fewer substances were commented on in the KEGG pathway. Based on the analysis of the substance structure, some substances detected in the sample, but not annotated on the KEGG pathway, were added to the corresponding pathway. This part of the material was not reflected on the KEGG pathway diagram, but the significance of KEGG was improved.

#### 4.7.8. Statistical Analysis

All physiological experiments were repeated at least three times independently. Cluster analysis, PCA and OPLS−DA were carried out using R, in accordance with previously described methods [73].

### 4.8. Quantitative Real-Time PCR Analysis 

To acquire insight into the molecular basis of flavonoid biosynthesis between plants in different cultivation methods (soil culture and nutrient solution culture), eight structural genes in the flavonoid pathway were identified from the *O. javanica* transcriptome database [12]. The total RNA of *O. javanica* cultivated with different methods was extracted using a plant RNA Extraction Kit (Tiangen, Beijing, China), and then reversed-transcribed into cDNA using HiScript^®^ IIl RT SuperMixfor qPCR (Vazyme, Nanjing, China). Key structural genes in the flavonoid pathway of *O. javanica* were selected for quantitative reverse−transcription polymerase chain reaction (qRT-PCR) analysis. qRT-PCR was performed in 20 μL reactions, consisting of 10 μL 2× ChamQ SYBR qPCR Master Mix (Vazyme, NanJing, China), 0.4 μL of a mixture of the forward and reverse primers, 1.0 μL cDNA template, and 8.2 μL ddH_2_O. The primer sequences for qRT-PCR assay were designed by the Primer 6 software [38]. *OjActin* gene was used as an internal standard to normalize the expression levels in *O. javanica* [74]. The amplifications were performed on a CFX−96 real-time PCR system (Bio−Rad), using the following quantitative real−time PCR program: 95 °C for 30 s, followed by 40 cycles of 95 °C for 10 s and 60 °C for 30 s. Each amplification reaction was performed in triplicate. The relative expression value of qRT-PCR assay was calculated with the 2^−ΔΔCT^ method [74].

## 5. Conclusions

In this study, the growth and quality of water dropwort cultivated in different nutrient solution formulations were detected. The results indicated that the Yamasaki nutrient solution was the best formula for the nutrient solution cultivation of water dropwort. The broadly targeted metabolomics further confirmed that the accumulation of metabolites under the Yamasaki nutrient liquid culture was significantly higher than that in soil culture. Flavonoids were investigated to be the most abundant differential metabolites under different cultivation methods. The structural genes in flavonoid biosynthesis pathway were significantly up−regulated under the Yamasaki nutrient liquid culture. These results provided a reference for a theoretical basis and technical guidance for the nutrient solution cultivation of water dropwort.

## Figures and Tables

**Table 1:** The effects of nutrient solution culture with different formulations on the growth characteristics of water dropwort.

Treatments	Plant Height (cm)	Root Length (cm)	Water Content (%)	Distribution Rate of Dry Matter (%)	Yield (g)
Hoagland (CK)	29.03 ± 2.17 ^a^	17.83 ± 0.80 ^c^	92.29 ± 1.38 ^a^	78.98 ± 0.27 ^a^	304.57 ± 12.48 ^ab^
Cooper	30.57 ± 2.06 ^a^	29.48 ± 0.95 ^a^	92.62 ± 0.63 ^a^	77.89 ± 1.18 ^a^	299.72 ± 40.38 ^ab^
Dutch greenhouse	30.45 ± 1.01 ^a^	25.82 ± 2.76 ^ab^	92.28 ± 0.31 ^a^	79.17 ± 0.24 ^a^	329.61 ± 15.63 ^a^
Garden-style	27.63 ± 3.46 ^a^	14.05 ± 2.01 ^c^	92.10 ± 0.72 ^a^	75.06 ± 3.41 ^ab^	264.88 ± 39.11 ^bc^
Yamasaki	31.18 ± 2.18 ^a^	16.51 ± 1.81 ^c^	92.96 ± 0.55 ^a^	75.19 ± 1.31 ^ab^	300.12 ± 6.70 ^ab^
SCAU	26.83 ± 4.42 ^a^	23.42 ± 3.92 ^b^	92.35 ± 0.24 ^a^	72.82 ± 3.51 ^b^	242.30 ± 36.79 ^c^

Values in the same column with different letters are significantly different (*p* < 0.05). Data are presented as the mean ± SD of three biological replicates.

**Table 2:** The effects of nutrient solution culture with different formulations on the quality characteristics of water dropwort.

Treatments	Chlorophyll (SPAD)	VC (μg/g)	Flavonoid (mg/g FW)	Total Phenolic (mg/g FW)	DPPH (%)	IDF (%)	SDF (%)	TDF (%)
Hoagland	45.00 ± 0.32 ^a^	43.35 ± 2.88 ^a^	1.52 ± 0.21 ^a^	8.88 ± 0.28 ^b^	68.18 ± 5.48 ^a^	55.22 ± 0.51 ^a^	6.10 ± 1.26 ^b^	61.32 ± 1.76 ^a^
Cooper	45.87 ± 1.24 ^a^	40.33 ± 7.60 ^a^	1.52 ± 0.12 ^a^	8.56 ± 0.77 ^b^	66.67 ± 6.80 ^a^	55.18 ± 1.08 ^a^	6.57 ± 1.86 ^ab^	61.75 ± 2.56 ^a^
Dutch greenhouse	45.19 ± 0.28 ^a^	42.16 ± 3.02 ^a^	1.61 ± 0.09 ^a^	9.61 ± 0.84 ^ab^	58.44 ± 8.22 ^a^	55.67 ± 1.27 ^a^	7.70 ± 1.08 ^ab^	63.37 ± 2.35 ^a^
Garden-style	44.91 ± 2.15 ^a^	46.94 ± 5.03 ^a^	1.66 ± 0.14 ^a^	9.44 ± 1.42 ^ab^	63.51 ± 4.63 ^a^	55.43 ± 0.96 ^a^	7.32 ± 1.71 ^ab^	62.75 ± 2.65 ^a^
Yamasaki	45.91 ± 0.76 ^a^	41.90 ± 3.25 ^a^	1.54 ± 0.2 1 ^a^	9.21 ± 1.10 ^b^	61.45 ± 1.19 ^a^	55.55 ± 1.65 ^a^	7.07 ± 0.45 ^ab^	62.62 ± 1.89 ^a^
SCUA	44.52 ± 2.22 ^a^	43.43 ± 2.46 ^a^	1.73 ± 0.26 ^a^	11.51 ± 1.87 ^a^	57.48 ± 9.20 ^a^	54.17 ± 3.71 ^a^	8.85 ± 1.10 ^a^	63.02 ± 4.10 ^a^

Values in the same column with different letters are significantly different (*p* < 0.05). VC: Vitamin C; DPPH: 1,1−diphenyl−2−picrylhydrazyl; IDF: Insoluble dietary fiber; SDF: Soluble dietary fiber; TDF: Total dietary fiber. Data are presented as the mean ± SD of three biological replicates.

**Table 3:** Comprehensive evaluation judgment matrix, weight value and identical test.

Hierarchy	Judgment Matrix					Weight Value	Identical Test
A-Ci	A	C1	C2				
	C1	1	1/4			0.2	CI = 0
	C2	4	1			0.8	CR = 0 < 0.1
C1-Pi	C1	P1	P2	P3	P4		
	P1	1	1/2	1/3	1/5	0.0838	CI = 0.0170
	P2	2	1	1/2	1/4	0.1377	CR = 0.0189 < 0.1
	P3	3	2	1	1/3	0.2323	
	P4	5	4	3	1	0.5462	
C2-Pi	C2	P5	P6	P7	P8		
	P5	1	1	1/3	1/4	0.1052	CI = 0.0278
	P6	1	1	1/3	1/4	0.1052	CR = 0.0309 < 0.1
	P7	3	3	1	1/3	0.2618	
	P8	4	4	3	1	0.5277	

**Table 4:** Final weight of indicator layer (P).

Indicators	P1	P2	P3	P4	P5	P6	P7	P8
Final weight	0.0168	0.0275	0.0465	0.1092	0.0842	0.0842	0.2094	0.4222

**Table 5:** Comprehensive evaluation value of different nutrient solution formula cultivation of water dropwort.

Treatments	Hoagland	Cooper	Dutch Greenhouse	Garden-Style	Yamasaki	SCUA
Comprehensive evaluation value	54.09	53.74	57.68	50.26	54.06	48.28

**Table 6:** Metabolite identification table of water dropwort.

Type	Number	Percentage (%)
All	485	100.0
Flavonoids	104	21.4
Phenolic acids	76	15.7
Amino acids and derivatives	61	12.6
Lipids	54	11.1
Organic acids	40	8.2
Nucleotides and derivatives	40	8.2
Lignans and Coumarins	30	6.2
Alkaloids	24	4.9
Terpenoids	2	0.4
Tannin	1	0.2
Others	53	10.9

## Data Availability

Not applicable.

## Supplementary Materials

The following supporting information can be downloaded at: https://www.mdpi.com/article/10.3390/plants12071459/s1, Table S1: Macronutrient formulae applied on soilless cultivation of water dropwort (mg/L); Figure S1: Sample quality evaluation of water dropwort with soil culture (CK) and Yamasaki nutrient solution (NK). (a) PCA score chart of test sample; (b) PCA score chart of test sample and quality control sample; Figure S2: Differential metabolite analysis on the basis of orthogonal signal correction and partial least squares−discriminant analysis (OPLS−DA). (a) The score chart. (b) verification chart; Figure S3: Primary classification of different metabolites of water dropwort cultivated in nutrient solution; Figure S4: QC sample mass spectrometry detection TIC current diagram.

## References

[1] Liu J.X., Jiang Q., Tao J.P., Feng K., Li T., Duan A.Q., Wang H., Xu Z.S., Liu H., Xiong A.S. (2021). Integrative genome, transcriptome, microRNA, and degradome analysis of water dropwort (*Oenanthe javanica*) in response to water stress. Hortic. Res..

[2] Park J.H., Cho J.H., Kim I.H., Ahn J.H., Lee J.C., Chen B.H., Shin B.N., Tae H.J., Yoo K.Y., Hong S. (2015). *Oenanthe Javanica* Extract Protects Against Experimentally Induced Ischemic Neuronal Damage via its Antioxidant Effects. Chin. Med. J..

[3] Kwon D., Yoon S., Carter O., Bailey G.S., Dashwood R.H. (2006). Antioxidant and antigenotoxic activities of *Angelica keiskei*, *Oenanthe javanica* and *Brassica oleracea* in the Salmonella mutagenicity assay and in HCT116 human colon cancer cells. BioFactors.

[4] Yang S.A., Jung Y.S., Lee S.J., Park S.C., Kim M.J., Lee E.J., Byun H.J., Jhee K.H., Lee S.P. (2014). Hepatoprotective effects of fermented field water-dropwort (*Oenanthe javanica*) extract and its major constituents. Food Chem. Toxicol..

[5] Ma C.J., Lee K.Y., Jeong E.J., Kim S.H., Park J., Choi Y.H., Kim Y.C., Sung S.H. (2010). Persicarin from water dropwort (*Oenanthe javanica*) protects primary cultured rat cortical cells from glutamate-induced neurotoxicity. Phytother. Res..

[6] Wang W.N., Yang X.B., Liu H.Z., Huang Z.M., Wu G.X. (2005). Effect of *Oenanthe javanica* flavone on human and duck hepatitis B virus infection. Acta Pharmacol. Sin..

[7] Feng K., Kan X.Y., Yan Y.J., Wang Y., Sun N., Yang Z.Y., Zhao S.P., Wu P., Li L.J. (2023). Identification and characterization of terpene synthase OjTPS1 involved in β-caryophyllene biosynthesis in *Oenanthe javanica* (Blume) DC. Ind. Crops Prod..

[8] Kumar S., Huang X., Li G., Ji Q., Zhou K., Zhu G., Ke W., Hou H., Zhu H., Yang J. (2021). Comparative Transcriptomic Analysis Provides Novel Insights into the Blanched Stem of *Oenanthe javanica*. Plants.

[9] Feng K., Kan X.Y., Li R., Yan Y.J., Zhao S.P., Wu P., Li L.J. (2022). Integrative Analysis of Long- and Short-Read Transcriptomes Identify the Regulation of Terpenoids Biosynthesis Under Shading Cultivation in *Oenanthe javanica*. Front. Genet..

[10] Song J.W., Shi Y.X., Xie X.W., Chai A.L., Li B.J. (2017). First report of grey mould on water dropwort (*Oenanthe javanica* DC.) caused by *Botrytis cinerea*. Can. J. Plant Pathol..

[11] Ji Q., Zhu H., Huang X., Zhou K., Liu Z., Sun Y., Wang Z., Ke W. (2021). Uncovering phylogenetic relationships and genetic diversity of water dropwort using phenotypic traits and SNP markers. PLoS ONE.

[12] Jiang Q., Wang F., Tan H.W., Li M.Y., Xu Z.S., Tan G.F., Xiong A.S. (2015). De novo transcriptome assembly, gene annotation, marker development, and miRNA potential target genes validation under abiotic stresses in *Oenanthe javanica*. Mol. Genet. Genom..

[13] Lu C.L., Li X.F. (2019). A Review of *Oenanthe javanica* (Blume) DC. as Traditional Medicinal Plant and Its Therapeutic Potential. Evid. Based Complement. Altern. Med..

[14] Amalfitano C., Vacchio L., Silvano S., Cuciniello A., Caruso G. (2017). Effects of cultural cycle and nutrient solution electrical conductivity on plant growth, yield and fruit quality of ‘Friariello’ pepper grown in hydroponics. Hortic. Sci..

[15] Lee J.Y., Rahman A., Azam H., Kim H.S., Kwon M.J. (2017). Characterizing nutrient uptake kinetics for efficient crop production during *Solanum lycopersicum* var. cerasiforme Alef. growth in a closed indoor hydroponic system. PLoS ONE.

[16] Savvas D., Samantouros K., Paralemos D., Vlachakos G., Stamatakis M., Vasilatos C. (2004). Yield and nutrient status in the root environment of tomatoes (*lycopersicon esculentum*) grown on chemically active and inactive inorganic substrates. Acta Hortic..

[17] Barbosa G.L., Gadelha F.D., Kublik N., Proctor A., Reichelm L., Weissinger E., Wohlleb G.M., Halden R.U. (2015). Comparison of Land, Water, and Energy Requirements of Lettuce Grown Using Hydroponic vs. Conventional Agricultural Methods. Int. J. Environ. Res. Public Health.

[18] Cifuentes-Torres L., Mendoza-Espinosa L., Reyes J.G., Daessle L. (2020). Hydroponics with wastewater: A review of trends and opportunities. Water Environ. J..

[19] Van Delden S.H., Nazarideljou M.J., Marcelis L.F.M. (2020). Nutrient solutions for *Arabidopsis thaliana*: A study on nutrient solution composition in hydroponics systems. Plant Methods.

[20] Zhang Y.Q., Xiao Z.L., Ager E., Kong L.Y., Tan L.B. (2021). Nutritional quality and health benefits of microgreens, a crop of modern agriculture. J. Future Foods.

[21] El-Nakhel C., Pannico A., Graziani G., Kyriacou M., Gaspari A., Ritieni A., De Pascale S., Rouphael Y. (2021). Nutrient Supplementation Configures the Bioactive Profile and Production Characteristics of Three Brassica Microgreens Species Grown in Peat-Based Media. Agronomy.

[22] Kumar S., Li G., Yang J., Huang X., Ji Q., Liu Z., Ke W., Hou H. (2021). Effect of Salt Stress on Growth, Physiological Parameters, and Ionic Concentration of Water Dropwort (*Oenanthe javanica*) Cultivars. Front. Plant Sci..

[23] Song J., Huang H., Hao Y., Song S., Zhang Y., Su W., Liu H. (2020). Nutritional quality, mineral and antioxidant content in lettuce affected by interaction of light intensity and nutrient solution concentration. Sci. Rep..

[24] Zhou X., Wang G. (2010). Nutrient concentration variations during *Oenanthe javanica* growth and decay in the ecological floating bed system. J. Environ. Sci..

[25] Tacnet J.M., Dezert J., Batton-Hubert M. (2011). AHP and Uncertainty Theories for Decision Making Using the ER-MCDA Methodology. International Symposium on Analytic Hierarchy/Network Process.

[26] Peng G., Han L., Liu Z., Guo Y., Yan J., Jia X. (2021). An Application of Fuzzy Analytic Hierarchy Process in Risk Evaluation Model. Front. Psychol..

[27] Upadhyay H.K., Juneja S., Muhammad G., Nauman A., Awad N.A. (2022). Analysis of IoT-Related Ergonomics-Based Healthcare Issues Using Analytic Hierarchy Process Methodology. Sensors.

[28] Bhushan N., Rai K., St C. (2022). Strategic Decision Making: Applying the Analytic Hierarchy Process (Decision Engineering).

[29] Ng C.Y., Chuah K. (2014). Evaluation of Design Alternatives’ Environmental Performance Using AHP and ER Approaches. IEEE Syst. J..

[30] Bino R.J., Hall R.D., Fiehn O., Kopka J., Saito K., Draper J., Nikolau B.J., Mendes P., Roessner-Tunali U., Beale M.H. (2004). Potential of metabolomics as a functional genomics tool. Trends Plant Sci..

[31] Fernie A.R. (2003). Review: Metabolome characterisation in plant system analysis. Funct. Plant Biol..

[32] Kopka J., Fernie A., Weckwerth W., Gibon Y., Stitt M. (2004). Metabolite profiling in plant biology: Platforms and destinations. Genome Biol..

[33] Oliver D.J., Nikolau B., Wurtele E.S. (2002). Functional genomics: High-throughput mRNA, protein, and metabolite analyses. Metab. Eng..

[34] Wu P., Liu A.L., Li L.J. (2021). Metabolomics and transcriptome analysis of the biosynthesis mechanism of flavonoids in the seeds of *Euryale ferox* Salisb at different developmental stages. Mol. Genet. Genom..

[35] Tiziani R., Miras-Moreno B., Malacrinò A., Vescio R., Lucini L., Mimmo T., Cesco S., Sorgonà A. (2022). Drought, heat, and their combination impact the root exudation patterns and rhizosphere microbiome in maize roots. Environ. Exp. Bot..

[36] Pelletier M.K., Burbulis I.E., Winkel-Shirley B. (1999). Disruption of specific flavonoid genes enhances the accumulation of flavonoid enzymes and end-products in Arabidopsis seedlings. Plant Mol. Biol..

[37] Deluc L., Barrieu F., Marchive C., Lauvergeat V., Decendit A., Richard T., Carde J.P., Mérillon J.M., Hamdi S. (2006). Characterization of a grapevine R2R3-MYB transcription factor that regulates the phenylpropanoid pathway. Plant Physiol..

[38] Feng K., Xu Z.S., Que F., Liu J.X., Wang F., Xiong A.S. (2018). An R2R3-MYB transcription factor, OjMYB1, functions in anthocyanin biosynthesis in *Oenanthe javanica*. Planta.

[39] Waraich E., Ahmad R., Ashraf M., Ullah S., Ahmad M. (2011). Improving agricultural water use efficiency by nutrient management in crop plants. Acta Agric. Scand. Sect. B Soil Plant Sci..

[40] Wang N., Fu F., Wang H., Wang P., He S., Shao H., Ni Z., Zhang X. (2021). Effects of irrigation and nitrogen on chlorophyll content, dry matter and nitrogen accumulation in sugar beet (*Beta vulgaris* L.). Sci. Rep..

[41] Hameed M.K., Umar W., Razzaq A., Aziz T., Maqsood M.A., Wei S., Niu Q., Huang D., Chang L. (2022). Differential Metabolic Responses of Lettuce Grown in Soil, Substrate and Hydroponic Cultivation Systems under NH(4)(+)/NO(3)(-) Application. Metabolites.

[42] Zhang M., Fang Y., Ji Y., Jiang Z., Wang L. (2013). Effects of salt stress on ion content, antioxidant enzymes and protein profile in different tissues of *Broussonetia papyrifera*. South Afr. J. Bot..

[43] Rahneshan Z., Nasibi F., Moghadam A.A. (2018). Effects of salinity stress on some growth, physiological, biochemical parameters and nutrients in two pistachio (*Pistacia vera* L.) rootstocks. J. Plant Interact..

[44] Singh M., Kumar J., Singh D.V., Prasad S. (2014). Plant Tolerance Mechanism Against Salt Stress: The Nutrient Management Approach. Biochem. Pharmacol..

[45] Lepiniec L., Debeaujon I., Routaboul J.M., Baudry A., Pourcel L., Nesi N., Caboche M. (2006). Genetics and biochemistry of seed flavonoids. Annu. Rev. Plant Biol..

[46] Tonga J.L., Kamdem M.H.K., Pagna J.I.M., Fonkui T.Y., Tata C.M., Fotsing M.C.D., Nkengfack E.A., Mmutlane E.M., Ndinteh D.T. (2022). Antibacterial activity of flavonoids and triterpenoids isolated from the stem bark and sap of *Staudtia kamerunensis* Warb. (Myristicaceae). Arab. J. Chem..

[47] Yang L.L., Zhou S.W., Hou Y.W., Ji B.Y., Pei L.X., Su X.H., Zhong H., Dong C.M. (2022). Blue light induces biosynthesis of flavonoids in *Epimedium sagittatum* (Sieb.et Zucc.) Maxim. leaves, a study on a light-demanding medicinal shade herb. Ind. Crops Prod..

[48] Treutter D. (2005). Significance of flavonoids in plant resistance and enhancement of their biosynthesis. Plant Biol..

[49] Routaboul J.M., Dubos C., Beck G., Marquis C., Bidzinski P., Loudet O., Lepiniec L. (2012). Metabolite profiling and quantitative genetics of natural variation for flavonoids in Arabidopsis. J. Exp. Bot..

[50] Maloney G.S., DiNapoli K.T., Muday G.K. (2014). The anthocyanin reduced tomato mutant demonstrates the role of flavonols in tomato lateral root and root hair development. Plant Physiol..

[51] Ren W., Qiao Z., Wang H., Zhu L., Zhang L. (2003). Flavonoids: Promising anticancer agents. Med. Res. Rev..

[52] Yao L.H., Jiang Y.M., Shi J., Tomás-Barberán F.A., Datta N., Singanusong R., Chen S.S. (2004). Flavonoids in food and their health benefits. Plant Foods Hum. Nutr..

[53] Cushnie T.P., Lamb A.J. (2005). Antimicrobial activity of flavonoids. Int. J. Antimicrob. Agents.

[54] Falcone Ferreyra M.L., Rius S.P., Casati P. (2012). Flavonoids: Biosynthesis, biological functions, and biotechnological applications. Front. Plant Sci..

[55] Wanner L.A., Li G., Ware D., Somssich I.E., Davis K.R. (1995). The phenylalanine ammonia-lyase gene family in Arabidopsis thaliana. Plant Mol. Biol..

[56] Austin M.B., Noel J.P. (2003). The chalcone synthase superfamily of type III polyketide synthases. Nat. Prod. Rep..

[57] Pang Y.Z., Shen G.A., Wu W.S., Liu X.F., Lin J., Tan F., Sun X.F., Tang K.X. (2005). Characterization and expression of chalcone synthase gene from *Ginkgo biloba*. Plant Sci..

[58] Sun W., Meng X., Liang L., Jiang W., Huang Y., He J., Hu H., Almqvist J., Gao X., Wang L. (2015). Molecular and Biochemical Analysis of Chalcone Synthase from Freesia hybrid in flavonoid biosynthetic pathway. PLoS ONE.

[59] Koes R.E., Quattrocchio F., Mol J.N.M. (1994). The flavonoid biosynthetic pathway in plants: Function and evolution. BioEssays.

[60] Tholl D. (2015). Biosynthesis and biological functions of terpenoids in plants. Adv. Biochem. Eng. Biotechnol..

[61] Li H.Z., Cheng Z.H. (2015). Hoagland nutrient solution promotes the growth of cucumber seedlings under light-emitting diode light. Acta Agric. Scand. Sect. B Soil Plant Sci..

[62] Tang Y., Fang Z., Liu M., Zhao D., Tao J. (2020). Color characteristics, pigment accumulation and biosynthetic analyses of leaf color variation in herbaceous peony (*Paeonia lactiflora* Pall.). 3 Biotech.

[63] Winkel-Shirley B. (2002). Biosynthesis of flavonoids and effects of stress. Curr. Opin. Plant Biol..

[64] Chen W., Gong L., Guo Z., Wang W., Zhang H., Liu X., Yu S., Xiong L., Luo J. (2013). A novel integrated method for large-scale detection, identification, and quantification of widely targeted metabolites: Application in the study of rice metabolomics. Mol. Plant.

[65] Wishart D.S., Jewison T., Guo A.C., Wilson M., Knox C., Liu Y., Djoumbou Y., Mandal R., Aziat F., Dong E. (2013). HMDB 3.0—The Human Metabolome Database in 2013. Nucleic Acids Res..

[66] Zhu G., Wang S., Huang Z., Zhang S., Liao Q., Zhang C., Lin T., Qin M., Peng M., Yang C. (2018). Rewiring of the Fruit Metabolome in Tomato Breeding. Cell.

[67] Zhu Z.J., Schultz A.W., Wang J., Johnson C.H., Yannone S.M., Patti G.J., Siuzdak G. (2013). Liquid chromatography quadrupole time-of-flight mass spectrometry characterization of metabolites guided by the METLIN database. Nat. Protoc..

[68] Fraga C.G., Clowers B.H., Moore R.J., Zink E.M. (2010). Signature-discovery approach for sample matching of a nerve-agent precursor using liquid chromatography-mass spectrometry, XCMS, and chemometrics. Anal. Chem..

[69] Eriksson L., Johansson E., Kettaneh-Wold N., Trygg J., Wikstr C., Wold S. (2006). Multi- and Megavariate Data Analysis. Part I Basic Principles and Applications. Second revised and enlarged edition. Ume Swed. MKS Umetrics AB.

[70] Zou J.N., Yu H., Yu Q., Jin X.J., Cao L., Wang M.Y., Wang M.X., Ren C.Y., Zhang Y.X. (2021). Physiological and UPLC-MS/MS widely targeted metabolites mechanisms of alleviation of drought stress-induced soybean growth inhibition by melatonin. Ind. Crops Prod..

[71] Li Q., Song J. (2019). Analysis of widely targeted metabolites of the euhalophyte *Suaeda salsa* under saline conditions provides new insights into salt tolerance and nutritional value in halophytic species. BMC Plant Biol..

[72] Kanehisa M., Goto S. (2000). KEGG: Kyoto encyclopedia of genes and genomes. Nucleic Acids Res..

[73] Wang S., Tu H., Wan J., Chen W., Liu X., Luo J., Xu J., Zhang H. (2016). Spatio-temporal distribution and natural variation of metabolites in citrus fruits. Food Chem..

[74] Schmittgen T.D., Livak K.J. (2008). Analyzing real-time PCR data by the comparative C(T) method. Nat. Protoc..

