# Supplementary material for: Metabolomics Analysis Reveals Metabolites and Metabolic Pathways Involved in the Growth and Quality of Water Dropwort [Oenanthe javanica (Blume) DC.] under Nutrient Solution Culture
Source: Plants (Basel). 2023 Mar 27;12(7):1459. doi: 10.3390/plants12071459 (PMC10097307; doi:10.3390/plants12071459)
Supplement: Supplementary file 1 [file plants-12-01459-s001.zip › plants-2178840-supplementary.pdf]

Table S1 Macronutrient formulae applied on soilless cultivation of water dropwort (mg/L).

Formula	Nutrition contents								Salts in total
	$Ca(NO3)_2\cdot 4H_2O$	KNO_3	NH_4NO_3	$(NH_4)_2SO_4$	$NH_4H_2PO_4$	$MgSO_4\cdot 7H_2O$	KH_2PO_4	K_2SO_4	
Hoagland	945	607	–	–	115	493	–	–	2160
Cooper	1062	505	–	–	–		140	–	2445
Dutch greenhouse	886	303	–	33	–		204	218	1891
Garden-style	945	809	–	–	153	493	–	–	2400
Yamasaki	826	607	–	–	115	483	–	–	1382
SCAU	472	404	53	–	–	246	100	116	1272

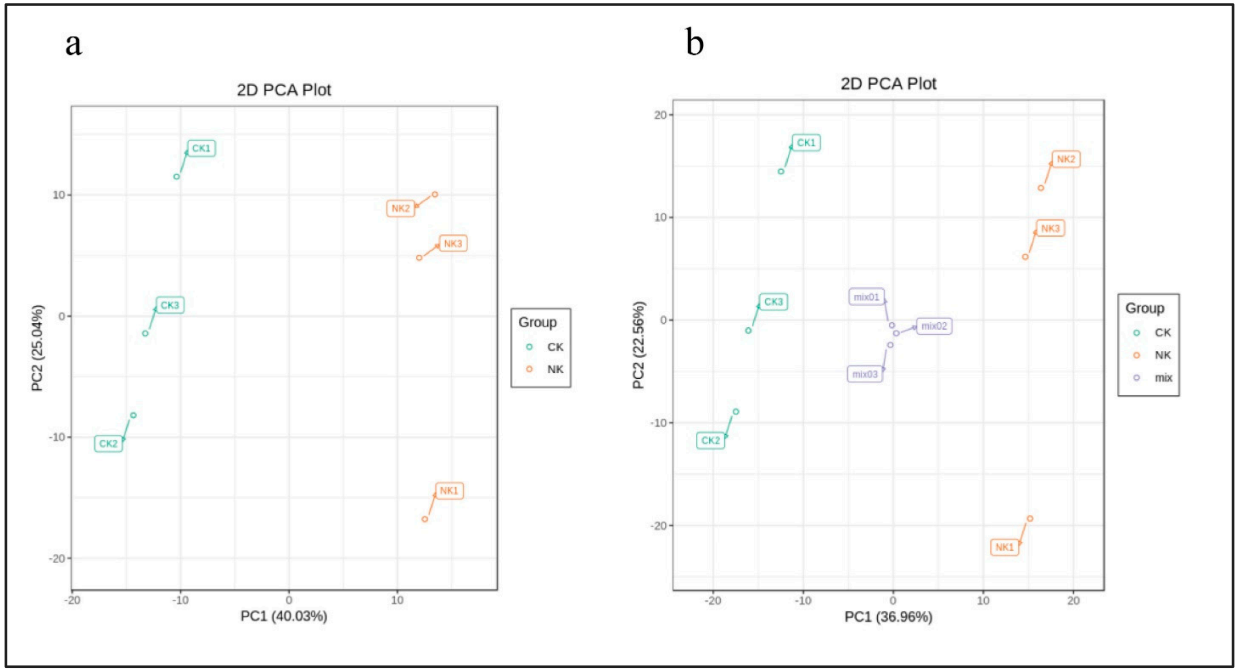

Figure S1. Sample quality evaluation of water dropwort with soil culture (CK) and Yamasaki nutrient solution (NK). (a) PCA score chart of test sample; **(b)** PCA score chart of test sample and quality control sample.

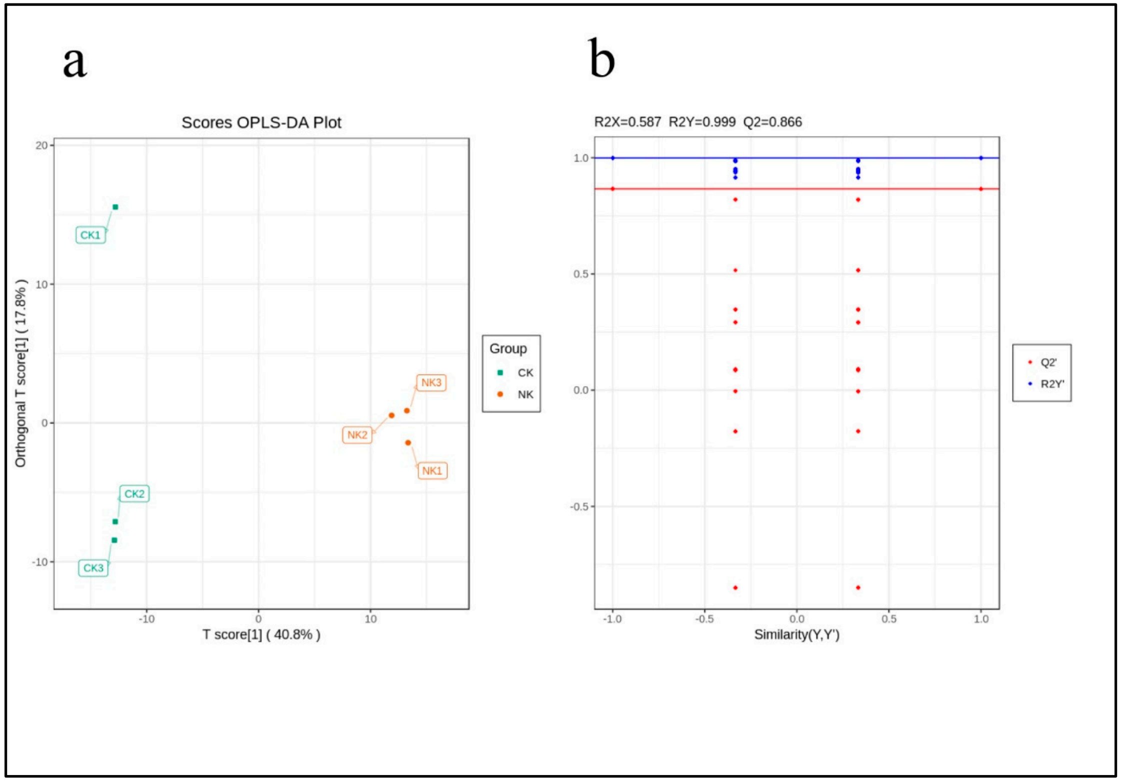

Figure S2. Differential metabolite analysis on the basis of orthogonal signal correction and partial least squares-discriminant analysis (OPLS-DA). (a) The score chart. **(b)** verification chart.

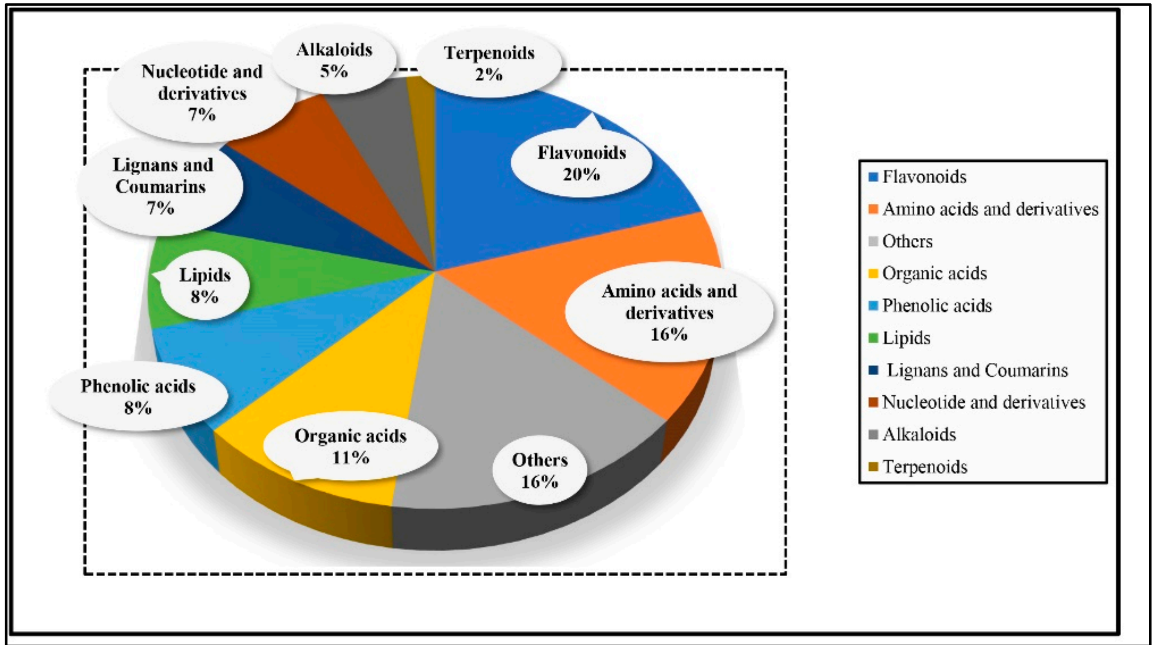

Figure S3. Primary classification of different metabolites of water dropwort cultivated in nutrient solution.

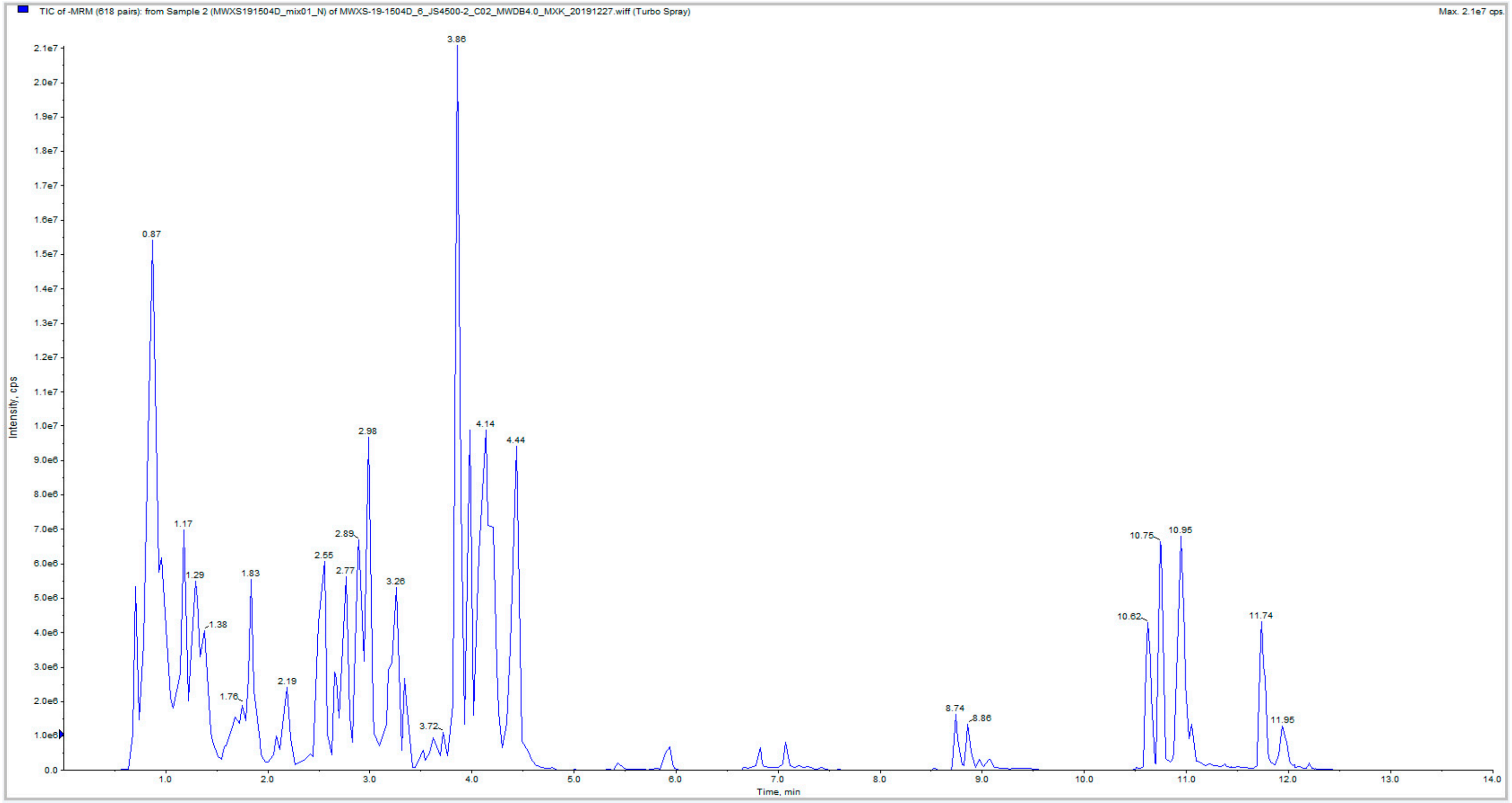

Figure S4. QC sample mass spectrometry detection TIC current diagram.
